# Nupr1/Chop signal axis is involved in mitochondrion-related endothelial cell apoptosis induced by methamphetamine

**DOI:** 10.1038/cddis.2016.67

**Published:** 2016-03-31

**Authors:** D Cai, E Huang, B Luo, Y Yang, F Zhang, C Liu, Z Lin, W-B Xie, H Wang

**Affiliations:** 1 Department of Forensic Medicine, School of Basic Medical Science, Southern Medical University, Guangzhou 510515, China; 2 Key Lab of Forensic Pathology, Guangdong Provincial Public Security Department, Guangzhou 510050, China; 3 Guangzhou Forensic Science Institute, Guangzhou 510030, China; 4 Department of Anatomy and Physiology, College of Veterinary Medicine, Institute of Computational Comparative Medicine, Kansas State University, Manhattan, KS 66506, USA

## Abstract

Methamphetamine (METH) abuse has been a serious global public health problem for decades. Previous studies have shown that METH causes detrimental effects on the nervous and cardiovascular systems. METH-induced cardiovascular toxicity has been, in part, attributed to its destructive effect on vascular endothelial cells. However, the underlying mechanism of METH-caused endothelium disruption has not been investigated systematically. In this study, we identified a novel pathway involved in endothelial cell apoptosis induced by METH. We demonstrated that exposure to METH caused mitochondrial apoptosis in human umbilical vein endothelial cells and rat cardiac microvascular endothelial cells *in vitro* as well as in rat cardiac endothelial cells *in vivo*. We found that METH mediated endothelial cell apoptosis through Nupr1–Chop/P53–PUMA/Beclin1 signaling pathway. Specifically, METH exposure increased the expression of Nupr1, Chop, P53 and PUMA. Elevated p53 expression raised up PUMA expression, which initiated mitochondrial apoptosis by downregulating antiapoptotic Bcl-2, followed by upregulation of proapoptotic Bax, resulting in translocation of cytochrome *c* (cyto *c*), an apoptogenic factor, from the mitochondria to cytoplasm and activation of caspase-dependent pathways. Interestingly, increased Beclin1, upregulated by Chop, formed a ternary complex with Bcl-2, thereby decreasing the dissociative Bcl-2. As a result, the ratio of dissociative Bcl-2 to Bax was also significantly decreased, which led to translocation of cyto *c* and initiated more drastic apoptosis. These findings were supported by data showing METH-induced apoptosis was significantly inhibited by silencing Nupr1, Chop or P53, or by PUMA or Beclin1 knockdown. Based on the present data, a novel mechanistic model of METH-induced endothelial cell toxicity is proposed. Collectively, these results highlight that the Nupr1–Chop/P53–PUMA/Beclin1 pathway is essential for mitochondrion-related METH-induced endothelial cell apoptosis and may be a potential therapeutic target for METH-caused cardiovascular toxicity. Future studies using knockout animal models are warranted to substantiate the present findings.

Methamphetamine (METH) is a widely used addictive stimulant with high potential of abuse. METH exposure damages both the nervous and cardiovascular systems.^1, 2, 3, 4, 5^ In particular, METH has been associated with a myriad of adverse effects on the circulatory system, including cardiomyopathy, hypertension, arrhythmia, myocardial ischemia, acute coronary syndrome, cardiac failure and sudden death.^6, 7, 8^ In METH abusers with acute aortic dissection or coronary syndrome, vascular structural alterations have been found in the myocardium in a number of clinical cases, indicating that METH can cause toxic effects on the blood vessels.^9, 10^ The above studies suggest that vascular endothelial cells may be a key target in METH-caused cardiovascular pathophysiologic alterations. Recent studies have shown that METH exposure causes endothelial cell apoptosis,^11, 12, 13^ but the underlying mechanisms remain to be elucidated.

Endoplasmic reticulum stress (ERS) pathway is a classical apoptotic pathway following the discovery of death receptor signaling and mitochondrial pathways.^14, 15^ In the present study, we hypothesized that Chop (as an ERS marker protein) is involved in endothelial cell apoptosis induced by METH. Chop (encoded by the *DDIT3* gene), is the key apoptosis inducer in the proteotoxic stress response.^16, 17^ Chop has been shown to be pro- and antiapoptotic depending on cell and stress context.^18^ Increased expression of the *DDIT3* gene or microinjections of the Chop protein led to dissipation of the mitochondrial transmembrane potential (MMP), generation of reactive oxygen species and apoptotic cell death.^19^

Recently, it was reported that increased expression of Chop and induction of apoptosis in response to ERS can be directly induced by nuclear protein 1 (Nupr1) in PANC-1 human pancreatic carcinoma cells.^20, 21^ It is known that Nupr1 (also named as p8 or com1) expression is upregulated in response to stress and thus influenced by the host microenvironment. Decreased Nupr1 expression is accompanied by suppression of cancer cell growth *in vitro* and *in vivo*.^22, 23^ However, increased Nupr1 mRNA also accompanies apoptotic changes in cancer cells.^24^ While *Nupr1* gene expression is induced in response to a variety of stress factors, *DDIT3* gene is specifically related to the ERS response.^25^

The objective of this study was to investigate the role of ERS and Nupr1 in METH-caused apoptosis in vascular endothelial cells. We determined METH-induced changes of Nupr1 expression and cellular apoptosis level *in vitro* using human umbilical vein endothelial cells (HUVECs) and rat cardiac microvascular endothelial cells (CMECs), as well as *in vivo* using vascular endothelium from Sprague–Dawley rats exposed to METH. Our results indicate that ERS induced by Nupr1 plays a crucial role in METH-induced vascular endothelial cell apoptosis and the Nupr1–Chop/P53–PUMA/Beclin1 pathway may be a potential therapeutic target of METH-induced cardiovascular toxicity.

## Results

### METH induces Nupr1 protein expression in vascular endothelial cells *in vivo* and *in vitro*

**Figure 1:**
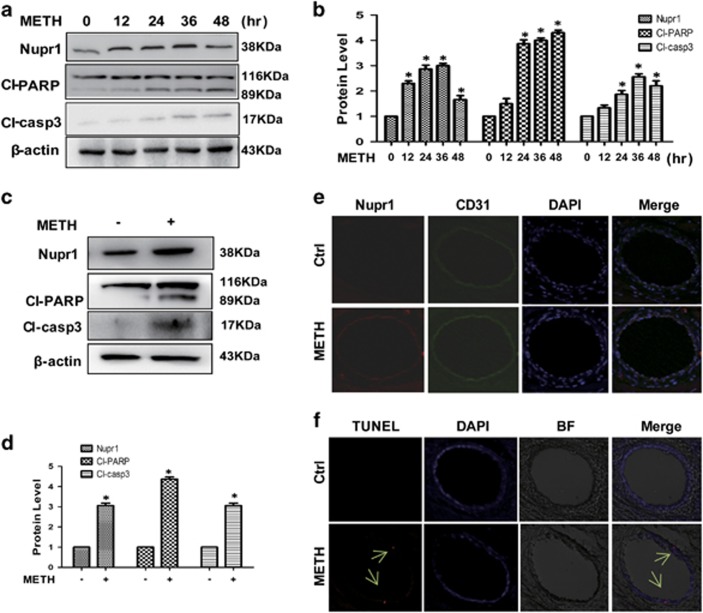
Nupr1 expression is upregulated in endothelial cells after METH exposure *in vitro* and *in vivo*. (**a**) HUVECs were exposed to 1.25 mM METH for indicated times (0, 12, 24, 36 and 48 h). (**c**) CMECs were exposed to METH (0.5 mM) for 24 h. The protein expression of Nupr1, cleaved-PARP and cleaved-caspase-3 was determined with western blot (**a** and **c**) and quantitative analyses (**b** and **d**). Fold induction relative to cells treated with vehicle is shown. β-Actin was used as a loading control. *P<0.05 versus vehicle-treated cells. Data were analyzed with one-way ANOVA followed by LSD *post hoc* comparisons. Data represent mean±S.D. (*n*=3 replicates). (**e** and **f**) Male SD rats (*n*=5/group) were injected i.p. with saline or METH (15 mg/kg × 8 injections, at 12 h interval). The heart tissues were harvested at 24 h after the last dosing. Immunolabeling and confocal imaging analysis (**e**) showed elevated Nupr1 expression in the heart microvascular endothelial cells of METH-exposed rats compared with controls (Ctrl). TUNEL staining and confocal imaging analysis (**f**) was used to evaluate the endothelial cell apoptosis. Apoptotic cells were stained with TUNEL (Red). Nuclei were counterstained with DAPI (blue) and BF represents bright field

To assess the role of Nupr1 in the METH-induced endothelial cell toxicity, HUVECs were exposed to 1.25 mM METH and then western blot analysis was performed to detect the expression of Nupr1. Our results revealed that Nupr1 expression was significantly increased (Figures 1a and b) in the METH-treated HUVECs than in the control. Cleaved-caspase-3 and cleaved-PARP (Figures 1a and b) were also significantly increased in the METH-treated HUVECs. Similar effects were observed in CMECs after METH (0.5 mM) exposure for 24 h (Figures 1c and d).

Furthermore, a rat model treated with METH (8 injections, 15 mg/kg/injection, at 12 h intervals) was used to ascertain whether METH induces Nupr1 expression and vascular endothelial cell apoptosis *in vivo*. Immunofluorescence staining results showed that METH treatment increased Nupr1 expression in rat cardiac microvascular endothelial tissues (Figure 1e). Apoptotic cells (labeled with arrows) in cardiac microvascular endothelial tissues were observed with TUNEL (terminal deoxynucleotidyl transferase (TdT)-mediated UTP nick end labeling) staining (Figure 1f). The results demonstrated that METH treatment caused microvascular endothelial cell apoptosis in rat cardiac tissues. Additionally, flow cytometry analyses showed that METH induced HUVECs and CMECs' apoptosis time dependently (Supplementary Figures S1a and b). These results suggest that METH exposure induces Nupr1 expression and endothelial cells' apoptosis both *in vivo* and *in vitro*.

### Nupr1 is necessary for METH-induced apoptosis in HUVECs and CMECs

**Figure 2:**
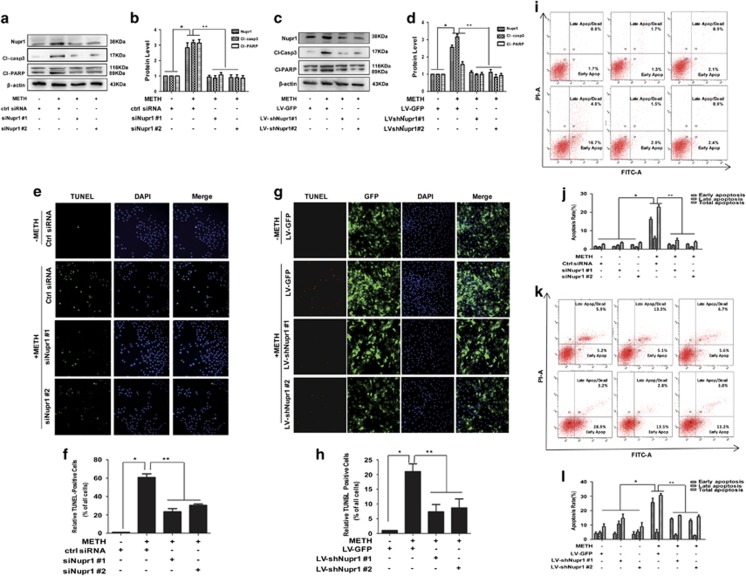
Nupr1 is necessary for METH-induced apoptosis in HUVECs and CMECs. (**a**) Synthetic Nupr1 siRNAs effectively suppressed endogenous Nupr1 expression on HUVECs. HUVECs were transfected with siRNAs targeting Nupr1 or control siRNA for 48 h followed by METH (1.25 mM) treatment for 24 h. (**c**) Lentivrus-mediated Nupr1 shRNAs effectively suppressed endogenous Nupr1 expression in CMECs. CMECs were incubated with viral supernatants for 24 h followed by METH (0.5 mM) treatment for another 24 h. Western blot (**a** and **c**) and quantitative analyses were performed to evaluate the efficiency of Nupr1 knockdown (**b** and **d**), and the expression of apoptosis-related proteins (cleaved-PARP (Cl-PARP) and cleaved-caspase-3 (Cl-casp3)) after knocking down Nupr1 expression in HUVECs (**b**) and CMECs (**d**). Effects of suppressing Nupr1 expression on the apoptosis caused by 1.25 mM METH in HUVECs and by 0.5 mM METH in CMECs were assessed with TUNEL (**e**: green for HUVECs; **g**: red for CMECs) and flow cytometry ((**i**) HUVECs and (**k**) CMECs). (**f** and **h**) Quantitative analysis of the percentage of apoptotic cells using a standard cell counting method with the TUNEL assay. Apoptotic cells were stained with TUNEL (green for HUVECs (**f**) and red for CMECs (**h**)). Nuclei were counterstained with DAPI (blue). (**j** and **l**) Quantitative analysis of the effects of knocking down Nupr1 on apoptosis induced by METH using flow cytometry. Representative calculations from three independent replicates are shown. The percentage of apoptotic cells is presented as mean± S.D. (*n*=3, *P<0.01 *versus* the saline vehicle-treated control group; **P<0.01 *versus* the scrambled+METH group; one-way ANOVA)

To examine whether Nupr1 is involved in the process of METH-induced apoptosis, we used siRNAs targeting Nupr1 to silence Nupr1 expression and then examined the effects on METH-caused apoptosis in HUVECs. Western blot analysis showed that both of two siRNAs can effectively knockdown Nupr1 expression (Figures 2a and b). Next, we evaluated whether silencing of Nupr1 by siRNAs also reduces METH-induced apoptosis *in vitro*. Western blot results showed that cleaved-caspase-3 and cleaved-PARP protein levels were decreased after Nupr1 knockdown in HUVECs (Figures 2a and b). Similar effects were observed in CMECs (Figures 2c and d).

To confirm that silence of Nupr1 protects against METH-induced apoptosis, TUNEL staining was performed (Figures 2e and f for HUVECs; Figures 2g and h for CMECs). These results suggest that blockade of Nupr1 expression reduces METH-induced apoptosis in HUVECs and CMECs, indicating that Nupr1 is involved in METH-induced apoptosis *in vitro*. To further evaluate the effect of Nupr1 on METH-caused toxicity, we investigated whether Nupr1 expression blockade affects apoptosis induced by METH in endothelial cells using flow cytometry analysis. Silence of Nupr1 significantly reduced the percentage of apoptotic cells caused by METH (Figures 2i and j). Similar results were obtained from CMECs (Figures 2k and l).

### Nupr1 mediates METH-induced endothelial cell apoptosis through the classical mitochondrial apoptotic signaling pathways

**Figure 3:**
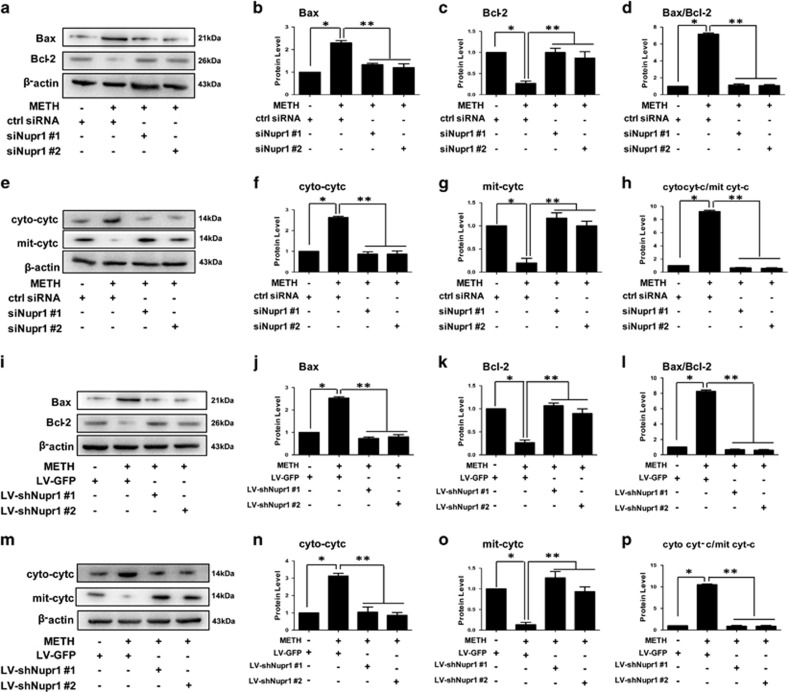
Nupr1 mediates METH-induced apoptosis through the classical mitochondrial apoptotic signaling pathways. (**a** and **e**) HUVECs were transfected with siRNAs targeting Nupr1 or control siRNA for 48 h followed by METH (1.25 mM) treatment for 24 h. (**i** and **m**) CMECs were incubated with LV-shRNA or LV-GFP control viral supernatants for 24 h followed by METH (0.5 mM) treatment for 24 h. The protein levels of Bax (**b** and **j**), Bcl-2 (**c** and **k**), cytosolic cyto *c* (cyto cyt-*c*; **f** and **n**), and mitochondrial cyto *c* (mit cyt-*c*; **g** and **o**) were measured using western blot analyses. The Bax/Bcl-2 (**d** and **l**) and cyto cyt-c/mit cyt-c (**h** and **p**) ratios were calculated. Data are presented as mean ±S.D. (*n*=3). *P<0.01 *versus* the saline vehicle-treated ctrl group, **P<0.01 *versus* the scrambled+METH group (one-way ANOVA)

To determine whether Nupr1 is involved in the mitochondria-dependent apoptosis induced by METH in endothelial cells, we examined the change of Bax and Bcl-2 expression level with and without Nupr1 knockdown. Western blot results showed that the expression level of Bax, a proapoptotic factor, was increased after exposure to METH for 24 h, and decreased following Nupr1 knockdown in HUVECs (Figures 3a and b), while the expression level of Bcl-2, an antiapoptotic factor, was decreased after exposure to METH for 24 h, and increased following Nupr1 knockdown in HUVECs (Figures 3a and c). The balance between proapoptotic proteins (e.g., Bax) and antiapoptotic proteins (e.g., Bcl-2) of Bcl family plays a key role in regulation of intrinsic pathway of cell apoptosis. In fact, the ratio of Bax/Bcl-2 was increased in METH-treated group compared with the ctrl group and decreased after Nupr1 was knocked down (Figure 3d). Similar results were observed in CMECs (Figures 3i–l).

We found that the cyto *c* protein level in cytoplasmic fraction was increased significantly when exposed to METH, while mitochondrial fraction was reduced significantly in HUVECs. This phenomenon was restrained after Nupr1 knockdown (Figures 3e–h). Similar results were obtained from CMECs (Figures 3m–p). Taken together, these results indicate that downregulation of Nupr1 inhibits the mitochondria-mediated apoptotic pathway induced by METH.

### Chop is involved in Nupr1-mediated apoptosis in METH-exposed endothelial cells

**Figure 4:**
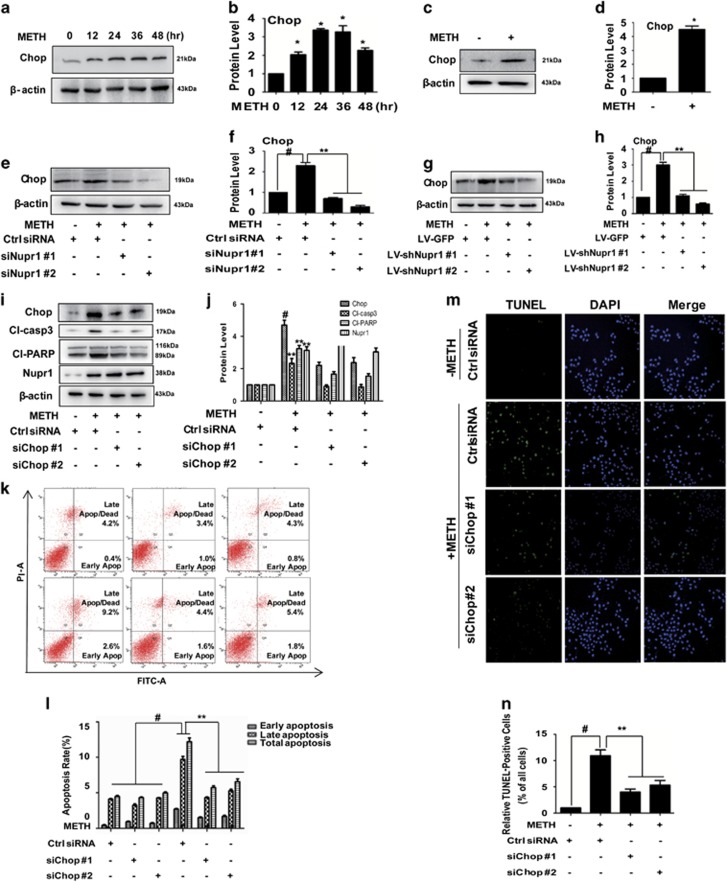
Chop is involved in Nupr1-mediated apoptosis in METH-exposed endothelial cells. (**a**) HUVECs were exposed to 1.25 mM METH for indicated time (0, 12, 24, 36 and 48 h). (**c**) CMECs were exposed to METH (0.5 mM) for 24 h. (**e**) HUVECs were transfected with siRNAs targeting Nupr1 or ctrl siRNA for 48 h followed by METH (1.25 mM) treatment for 24 h. (**g**) CMECs were incubated with LV-shNupr1 or LV-GFP control viral supernatants for 24 h followed by METH (0.5 mM) treatment for 24 h. (i) HUVECs were transfected with siRNAs targeting Chop or ctrl siRNA for 48 h followed by METH (1.25 mM) treatment for 24 h. Western blot (**a**, **c**, **e**, **g**, **i**) and quantitative analyses (**b**, **d**, **f**, **h** and **j**) were performed to evaluate the expression of Chop, Nupr1, cleaved-PARP (Cl-PARP) and cleaved-caspase-3 (Cl-casp3). Effects of suppressing Chop expression on 1.25 mM METH-treated HUVECs were assessed by flow cytometry (**k** and **l**) and TUNEL (**m** and **n**). Apoptotic cells were stained with TUNEL (green). Nuclei were counterstained with DAPI (blue). (**l**) Quantitative analysis of the effects of Chop knockdown on apoptosis induced by METH using flow cytometry. (**n**) Quantitative analysis of the percentage of apoptotic cells using a standard cell counting method with the TUNEL assay. Representative calculations from three independent experiments are shown. The rate of apoptosis is presented as mean±S.D. (*n*=3, *P<0.01 *versus* the saline vehicle-treated ctrl group, **P<0.01 *versus* the scrambled+METH group, one-way ANOVA)

Chop, a transcription factor, is a key mediator of cell death in response to ERS. In the present study, we found that Chop protein expression was increased in both HUVECs (Figures 4a and b) and CMECs (Figures 4c and d) after METH exposure. Increased expression of Chop and induction of apoptosis in response to ERS have been associated with Nupr1 activation in astrocytoma cells exposed to cannabinoid,^26^ so we assessed the effect on Chop expression after silencing Nupr1. We observed that Chop expression was decreased following Nupr1 expression knockdown in METH-treated HUVECs (Figures 4e and f) and CMECs (Figures 4g and h).

Next, we examined whether Chop is involved in METH-induced apoptosis. We found that METH exposure induced Chop expression in the ctrl siRNA group; this effect was significantly attenuated by co-exposure to either of the siChop sequences (Figures 4i and j). The expression level of cleaved-caspase-3 and cleaved-PARP were decreased following Chop knockdown in HUVECs (Figures 4i and j). However, the expression of Nupr1 showed no difference, indicating that Chop does not regulate Nupr1 (Figures 4i and j). Similar effects were observed in CMECs (Supplementary Figures S2a and c). Additionally, we observed that Bax was increased after METH treatment and decreased following Chop knockdown in CMECs (Supplementary Figures S2a and d), while the expression level of Bcl-2 was decreased after METH exposure and increased following Chop knockdown in CMECs (Supplementary Figures S2a and e). The ratio of Bax/Bcl-2 was increased in the METH-treated group compared with the ctrl group and decreased after Chop knock down in CMECs (Supplementary Figure S2f).

We also investigated whether the Chop expression blockade affects METH-induced apoptosis in HUVECs using flow cytometry analysis (Figures 4k and l) and TUNEL staining (Figures 4m and n). These results indicate that the blockade of Chop expression also inhibits endothelial cells' apoptosis induced by METH.

### Nupr1/Chop/P53 axis is involved in classical mitochondrial apoptosis caused by METH in endothelial cells

**Figure 5:**
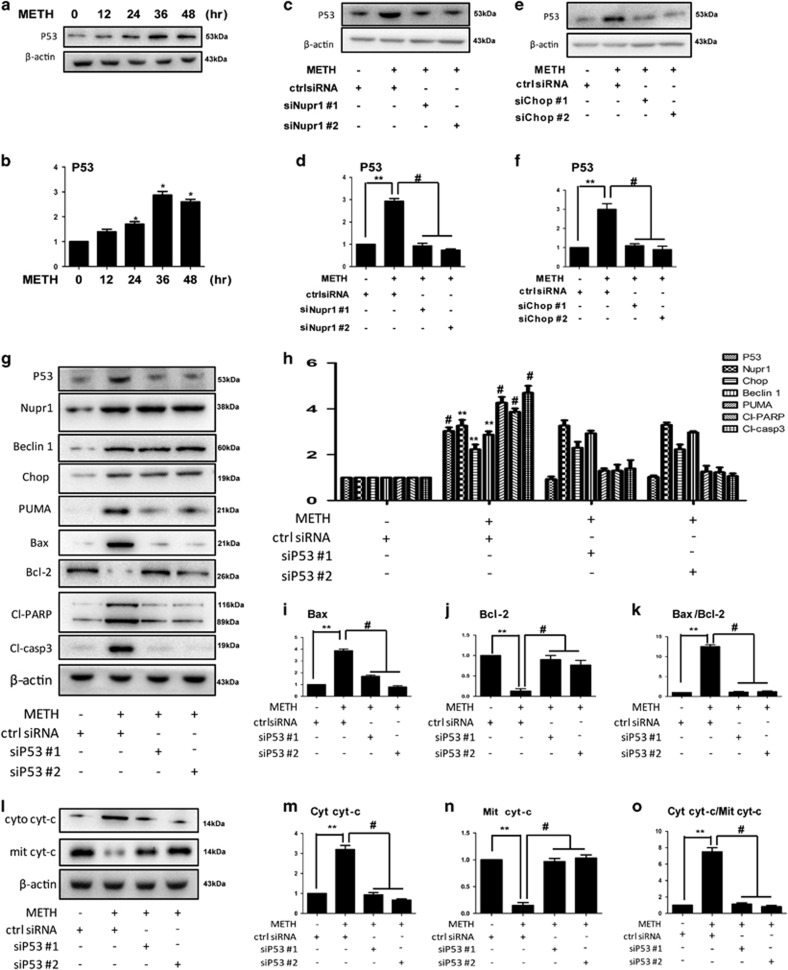
P53 is involved in Nupr1–Chop axis-mediated mitochondrial apoptotic signaling pathways caused by METH in endothelial cells. (**a**) HUVECs were exposed to METH (1.25 mM) for 24 h. (**c**, **e** and **g**) HUVECs were transfected with siRNAs targeting Nupr1 (**c**), Chop (**e**), P53 (**g**) or ctrl siRNA for 48 h followed by METH (1.25 mM) treatment for 24 h. Western blot (**a**, **c**, **e**, **g**) and quantitative analyses (**b**, **d**, **f** and **h**–**j**) were performed to evaluate the expression of P53, Beclin1, Bax, Bcl-2, Chop, Nupr1, cleaved-PARP (Cl-PARP) and cleaved-caspase-3 (Cl-casp3). The protein levels of cytosolic cyto *c* and mitochondrial cyto *c* (**l**–**n**) were measured using western blot analyses. The Bax/Bcl-2 (**k**) and cyto cyt-*c*/mit cyt-*c* (**o**) ratios were calculated. Data are presented as mean ±S.D. (*n*=3). *P<0.01 *versus* the saline vehicle-treated ctrl group, **P<0.01 *versus* the scrambled+METH group (one-way ANOVA)

The downstream targets of Chop in Nupr1–Chop axis-mediated METH-induced endothelia cell apoptosis have not been identified. Among many downstream targets of Chop, the P53 protein is a key regulator of cell cycle, apoptosis, DNA repair and senescence.^27, 28^ Next, we evaluated the role of p53 in METH-induced endothelial cell apoptosis. We found that METH exposure increased P53 expression significantly in both HUVECs (Figures 5a and b) and CMECs (Supplementary Figures S3a and b). P53 expression was decreased following Nupr1 or Chop knockdown in METH-treated HUVECs, suggesting that P53 is regulated by Nupr1 and Chop in HUVECs (Figures 5c–f). We also observed this phenomenon in METH-treated CMECs (Supplementary Figures S3c–f), which indicates that P53 is the downstream protein of Nupr1 and Chop. On the other hand, Nupr1 and Chop expression did not change following P53 knockdown in METH-treated HUVECs (Figures 5g and h) and CMECs (Supplementary Figures S3g and h), suggesting that both Nupr1 and Chop is not regulated by p53. Instead, Nupr1 regulates P53 in METH-treated HUVECs and CMECs.

Furthermore, we found that cleaved-caspase-3 and cleaved-PARP protein levels were decreased following P53 expression knockdown in HUVECs (Figures 5g and h) and CMECs (Supplementary Figures S3g and h). The expression level of Bax was increased after METH exposure and decreased following P53 knockdown, while Bcl-2 was decreased after METH exposure and increased following P53 knockdown in HUVECs (Figures 5g, i and j) and CMECs (Supplementary Figures S3g, i and j). The ratio of Bax/Bcl-2 was increased in METH-treated group compared with the ctrl group and decreased after P53 knockdown in HUVECs (Figure 5k) and CMECs (Supplementary Figure S3k). We also found that cyto *c* protein was translocated from mitochondria to cytoplasm and that the translocation was reversed by silence of P53 in HUVECs (Figures 5l–o) and CMECs (Supplementary Figures 3l–o). Taken together, these results indicate that P53 is involved in Nupr1–Chop axis-mediated METH-induced mitochondrial apoptosis in endothelial cells.

### PUMA is the initiator in the Nupr1/Chop axis-activated classical mitochondrial apoptotic signaling pathways in METH-exposed endothelial cells

**Figure 6:**
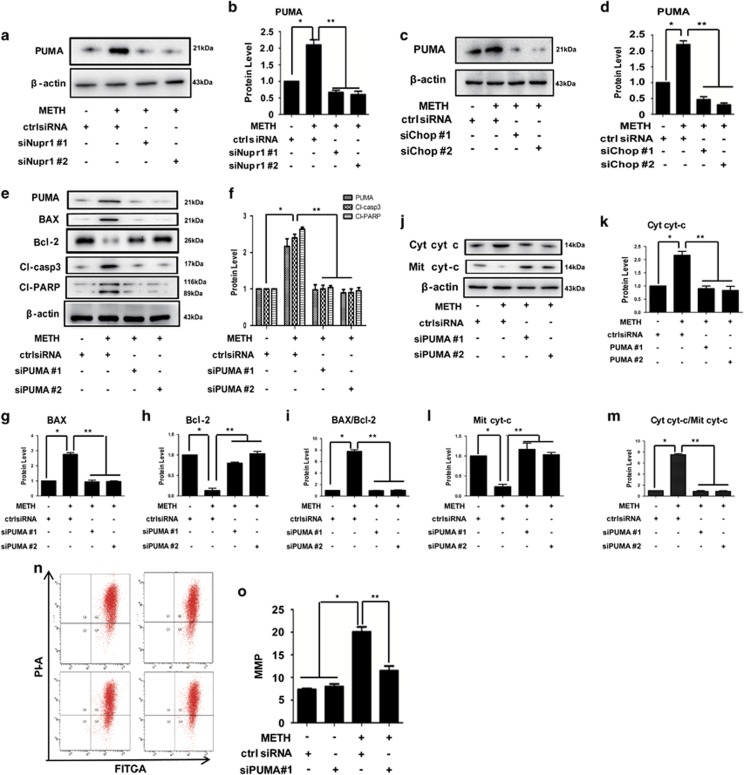
PUMA is the initiator in the Nupr1/Chop axis-activated classical mitochondria apoptotic signaling pathways. HUVECs were transfected with siRNAs targeting Nupr1 (**a**), Chop (**c**), PUMA (**e**) or ctrl siRNA for 48 h followed by METH (1.25 mM) treatment for 24 h. Western blot (**a**, **c**, **e**, **j**) and quantitative analyses (**b**, **d**, **f**, **g**–**i**, **k**–**m**) were performed to evaluate the expression of PUMA, Bax, Bcl-2, cleaved-PARP (Cl-PARP), cleaved-caspase-3 (Cl-casp3), the ratio of Bax/Bcl-2, cytosolic cyto *c* (cyt cyt-*c*), mitochondrial cyto *c* (Mit cyt-*c*) and the Cyt cyt-*c*/Mit cyt-*c* ratio. (**n**) Mitochondrial membrane potential (MMP) was analyzed with fluorescent probe JC-1 assay. (**o**) Quantitative results of JC-1 assay. Data are presented as mean±S.D. (*n*=3). *P<0.05 *versus* the saline vehicle-treated control group and **P<0.05 *versus* the scrambled+METH group (one-way ANOVA)

Previous studies have demonstrated that PUMA induces apoptosis in part by displacing Bax from Bcl-XL, thereby promoting the multimerization and mitochondrial translocation of Bax.^29, 30, 31^ To assess the role of PUMA in the METH-induced toxicity, HUVECs were treated with METH and then western blot analysis was performed to detect PUMA expression. The results showed that PUMA expression was significantly increased by METH exposure (Supplementary Figure S4a). Similar effects were observed in CMECs (Supplementary Figure 4b). Next, we studied the effect of Nupr1 knockdown on the increased expression of PUMA caused by METH. We found that PUMA expression was decreased following Nupr1 knockdown in METH-treated HUVECs, suggesting that PUMA is regulated by Nupr1 (Figures 6a and b). We also observed that PUMA expression was reduced after Chop knockdown in METH-treated HUVECs (Figures 6c and d), which indicates that PUMA is the downstream protein of Chop. In addition, we observed that PUMA expression was decreased following P53 knockdown in METH-exposed HUVECs (Figures 5g and h) and CMECs (Supplementary Figures S3g and h), suggesting that PUMA is downstream target of P53. As demonstrated previously, Chop was regulated by Nupr1 in METH-treated HUVECs (Figures 4e and f) and CMECs (Figures 4g and h), and P53 was regulated by Nupr1 and Chop in METH-treated HUVECs (Figures 5c–f) and CMECs (Supplementary Figures S3c–f). Based on these results, we hypothesized that Nupr1 regulates PUMA expression through Chop–P53 axis in METH-induced apoptosis process.

To test this hypothesis, we investigated that if PUMA is responsible for METH-induced apoptosis. Western blot analysis showed that METH exposure induced PUMA expression in the ctrl siRNA group and this effect was significantly attenuated by co-exposure to the siPUMA sequence (Figures 6e and f). We found that Bax was increased after 24 h METH exposure, and decreased following PUMA knockdown in HUVECs, while the expression level of Bcl-2 showed opposite effects (Figures 6e, g and h). The cytosolic cyto *c* protein level was increased significantly, while mitochondrial cyto *c* was decreased significantly when exposed to METH (Figures 6j–m). Together, these results suggest that silencing of PUMA expression reduces METH-induced apoptosis in HUVECs. Similar effects were observed in CMECs (Supplementary Figure S5).

To confirm that silence of PUMA expression protects against METH-induced apoptosis, we analyzed the changes in the MMP using the JC-1 indicator, as an independent method, to evaluate the induction of the proapoptotic process in METH-treated HUVECs. It was found that the METH-caused reduction of HUVEC viability was accompanied by a reduction in the transmembrane potential in approximately 20% of the cells. However, this reduction was significantly attenuated to 12% after PUMA knockdown (Figures 6n and o). All of these results indicate that PUMA plays a key role in the Nupr1/Chop axis-activated classical mitochondrial apoptotic signaling pathways in METH-exposed endothelial cells.

### Beclin1 promotes apoptosis by binding with Bcl-2 and through the Nupr1/Chop axis-activated classical mitochondria apoptotic signaling pathways in METH-exposed endothelial cell

**Figure 7:**
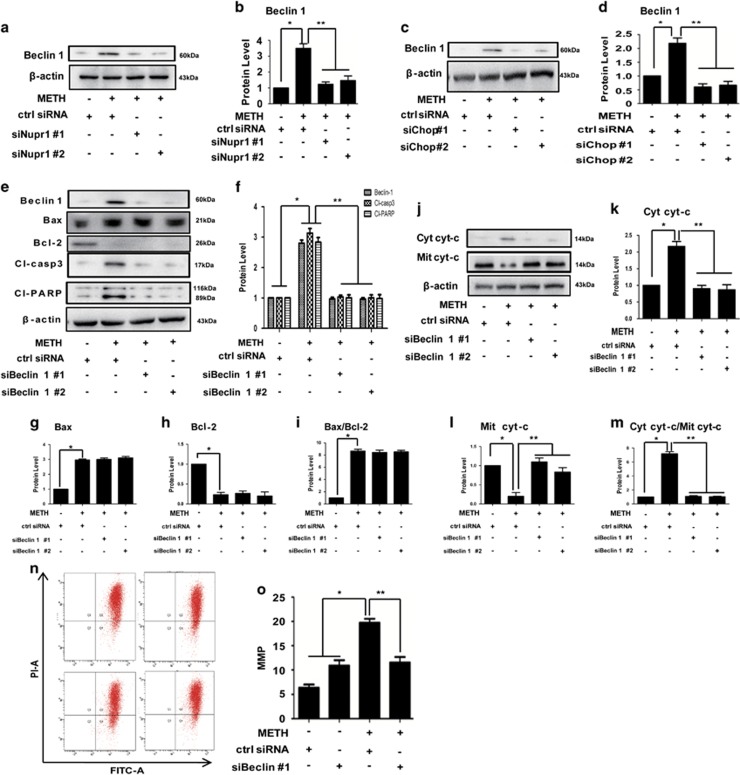
Beclin1 promotes apoptosis through the Nupr1/Chop axis-activated classical mitochondria apoptotic signaling pathways. HUVECs were transfected with siRNAs targeting Nupr1 (**a**), Chop (**c**), Beclin1 (**e**), or control siRNA for 48 h followed by METH (1.25 mM) treatment for 24 h. Western blot (**a**, **c**, **e** and **j**) and quantitative analyses (**b**, **d**, **f** and **g**–**i**, **k**–**m**) were performed to evaluate the expression of Beclin1, cleaved-PARP (Cl-PARP), cleaved-caspase-3 (Cl-casp3), Bax, Bcl-2, Bax/Bcl-2 ratio, cytosolic cyto *c* (Cyt cyt-*c*), mitochondrial cyto *c* (Mit cyt-*c*) and Cyt cyt-*c*/Mit cyt-*c* ratio. (**n**) Mitochondrial membrane potential (MMP) was analyzed with fluorescent probe JC-1 assay. (**o**) Quantitative results of JC-1 assay. Data are presented as mean±S.D. (*n*=3). *P<0.05 *versus* the saline vehicle-treated ctrl group and **P<0.05 *versus* the scrambled+METH group (one-way ANOVA)

Since Beclin1 is known to be involved in the signal crosstalk between ERS and mitochondrial dysfunction, our next question was if METH-induced apoptosis is mediated through Beclin1. We observed that Beclin1 expression was significantly increased after METH exposure in HUVECs (Supplementary Figures S6a and a1) and CMECs (Supplementary Figures S6b and b1). Notably, we observed that Beclin1 expression was decreased after Nupr1 knockdown in HUVECs (Figures 7a and b). These results suggest that Beclin1 is regulated by Nupr1. Besides Nupr1, we also observed that Beclin1 expression was reduced following Chop knockdown in HUVECs (Figures 7c and d) and in CMECs (Supplementary Figures S2a and b), indicating that Beclin1 is also regulated by Chop. However, our results showed that Beclin1 expression did not change following P53 knockdown in METH-treated HUVECs (Figures 5g and h) and CMECs (Supplementary Figures S3g and h), indicating that Beclin1 is not a downstream target of P53.

Previously, we demonstrated that Chop was regulated by Nupr1 (Figures 4e–h). Consequently, we further hypothesized that Nupr1 regulates Beclin1 expression through Chop in METH-induced apoptosis. Western blot analysis showed that METH exposure induced Beclin1 expression in the ctrl siRNA group; this effect was significantly mitigated by co-treatment with the siRNAs (Figures 7e and f). In addition, the expression level of cleaved-caspase-3 and cleaved-PARP was increased after METH exposure for 24 h, and decreased following Beclin1 knockdown in HUVECs (Figures 7e and f) and CMECs (Supplementary Figures S7a and b). We also observed that cyto *c* was translocated from mitochondria to cytoplasmic fraction after METH exposure in HUVECs (Figures 7j–m) and CMECs (Supplementary Figures S7c–f). Notably, silencing of Beclin1 expression had no effect on Bax and Bcl-2 expression induced by METH (Figures 7e, g–i), which indicates that Beclin1-mediated mitochondria-related apoptosis is not through regulating the expression of Bax and Bcl-2.

**Figure 8:**
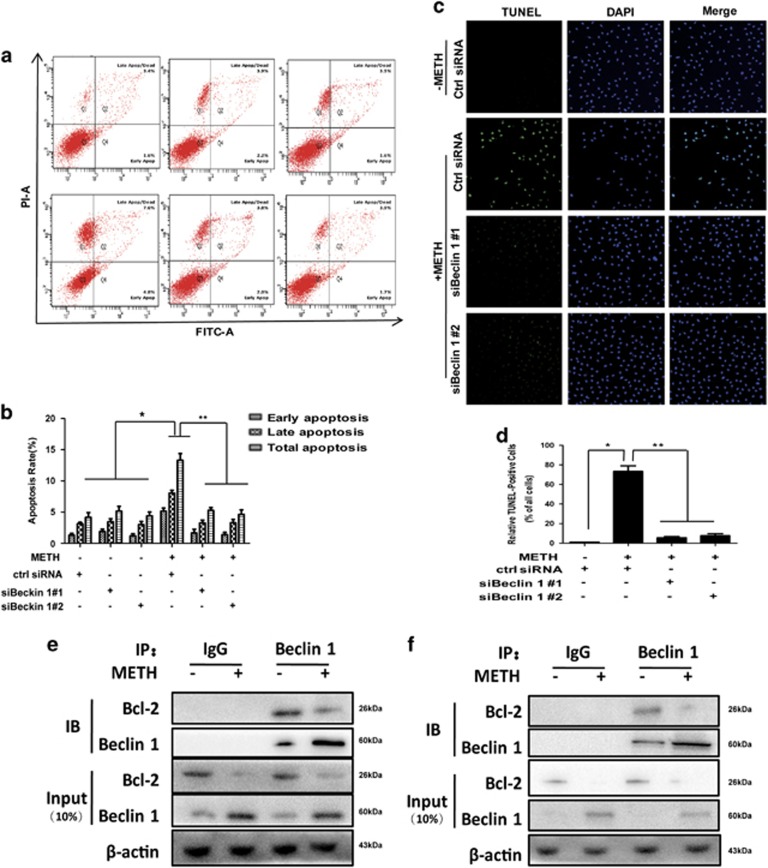
Beclin1 promotes apoptosis by physically interacting with Bcl-2. Effects of suppressing Beclin1 expression on the apoptosis caused by 1.25 mM METH in HUVECs were assessed with flow cytometry (**a**) and TUNEL (**c**: green for HUVECs). (**b**) Quantitative analysis of the effects of knocking down Beclin1 on apoptosis induced by METH using flow cytometry. (**d**) Quantitative analysis of the percentage of apoptotic cells using a standard cell counting method with the TUNEL assay. Nuclei were counterstained with DAPI (blue). (**e**) Endogenous Bcl-2 co-immunoprecipitated with Beclin1. Cell lysates from METH-treated HUVECs were immunoprecipitated with Beclin1 antibody or normal IgG and blotted with Bcl-2 antibody. (**f**) Endogenous Beclin1 co-immunoprecipitated with Bcl-2. Cell lysates from METH-treated CMECs were immunoprecipitated with Bcl-2 antibody or normal IgG and blotted with Beclin1 antibody. Representative calculations from three independent replicates are shown. The percentage of apoptotic cells is presented as mean±S.D. (*n*=3, *P<0.01 *versus* the saline vehicle-treated control group; **P<0.01 *versus* the scrambled+METH group; one-way ANOVA)

To confirm that silence of Beclin1 expression protects against METH-induced apoptosis, we analyzed the changes in the MMP using the JC-1 indicator. It was found that the reduction in HUVECs cell viability caused by METH was associated with a reduction in the MMP (Figures 7n and o). We also investigated whether silencing Beclin1 expression affects METH-induced apoptosis in HUVECs using flow cytometry analysis (Figures 8a and b) and TUNEL staining (Figures 8c and d). Silence of Beclin1 significantly reduced the percentage of apoptotic cells caused by METH exposure. Collectively, these results indicate that the blockade of Beclin1 expression inhibits endothelial cells' apoptosis induced by METH.

Since our results showed that Beclin1 mediated mitochondria-related METH-induced apoptosis not through regulating the protein expression of Bax and Bcl-2, we hypothesized that Beclin1 might regular METH-induced apoptosis by forming Bcl-2/Beclin1 complex. Next, we used the co-immunoprecipitation technique to test the hypothesis. As shown in Figures 8e and f, the interaction between Beclin1 and Bcl-2 was detected in METH-exposed HUVECs (Figure 8e) and CMECs (Figure 8f), suggesting that Beclin1 mediates mitochondria-related apoptosis is through interacting with Bcl-2, thereby decreasing the dissociative Bcl-2.

## Discussion

Recent studies have demonstrated that METH can impair the blood–brain barrier (BBB), suggesting that some of the neurotoxic effects of METH could be due to disruption of BBB, which includes vascular endothelial cells.^32^ In the present study, we show that METH exposure induces apoptosis of endothelial cells both *in vitro* and *in vivo*, which is consistent with the previous studies.^11, 33^ Furthermore, we for the first time demonstrate that ERS plays an essential role in METH-induced endothelial cell apoptosis and the Nupr1/Chop signal axis regulates the interaction between ERS and METH-caused mitochondria-depending apoptosis.

Nupr1 can regulate autophagy and apoptosis, and may be relevant to cardiovascular pathologies and cancers.^34^ Intriguingly, Nupr1 seems to play a dual role in the control of cell fate. Nupr1 has also been implicated in mediating cannabinoid-induced apoptosis of tumor cells through upregulation of the ERS-related genes *ATF4*, *Chop* and *Trib3* (tribbles pseudokinase 3). All these observations suggest that cell or tissue-specific factors may determine the final role played by Nupr1 in the control of cell fate.^35^ Here we report that Nupr1 mediates cell apoptosis induced by METH in endothelial cells. In addition, we identified Chop, a transcription factor of CCAAT/enhancer binding protein homologous protein family, which is strictly correlated with ERS and participates in ER-mediated apoptosis,^36^ as the downstream of Nupr1.

Chop is a non-ER localized transcription factor that is induced by a variety of adverse physiological conditions, including ERS, and it is thought to be a critical mediator of stress-induced apoptosis.^37, 38^ Chop was shown to bind to the PUMA promoter during ERS and Chop knockdown attenuated PUMA induction and neuronal apoptosis.^39, 40, 41, 42^ Here, we demonstrated that Chop increases the expression level of PUMA through P53 to mediate apoptosis caused by METH through the mitochondrial pathway, resulting in changes in the mitochondrial membrane potential, promotion of cyto *c* release and initiation of caspase cascades. Specifically, following METH exposure, Chop increases the expression of Bax and decreases the expression of Bcl-2, which leads to an increased Bax/Bcl-2 ratio, thereby inducing the release of cyto *c* to initiate caspase cascades. Of note, there are other downstream targets of Chop, including Bim (BH3-only protein). Further studies are needed to identify additional proteins that play a role in the Nupr1–Chop axis-mediated METH-induced endothelial cell apoptosis.

In our experiments, we showed that Beclin1 is involved in the mitochondrial apoptosis caused by METH. Notably, silencing of Beclin1 expression has no effect on Bax and Bcl-2 expression induced by METH, which indicates that Beclin1-mediated mitochondria-related apoptosis is not through regulating the expression of Bax and Bcl-2. There is no evidence that Beclin1 has another pathway to induce apoptosis other than forming Bcl-2/Beclin1 complex, which regulates both apoptosis and autophagy by controlling of the threshold between cell survival and cell death. Our co-immunoprecipitation results suggest that Beclin1 physically interacts with Bcl-2. Therefore, we conclude that Beclin1 mediates mitochondria-related apoptosis may be through forming a ternary complex with Bcl-2, thereby decreasing the dissociative Bcl-2. However, further experiments are necessary to fully understand the role of the Bcl-2/Bcl-xL–Beclin1 interaction in METH-induced apoptosis.^43, 44^

**Figure 9:**
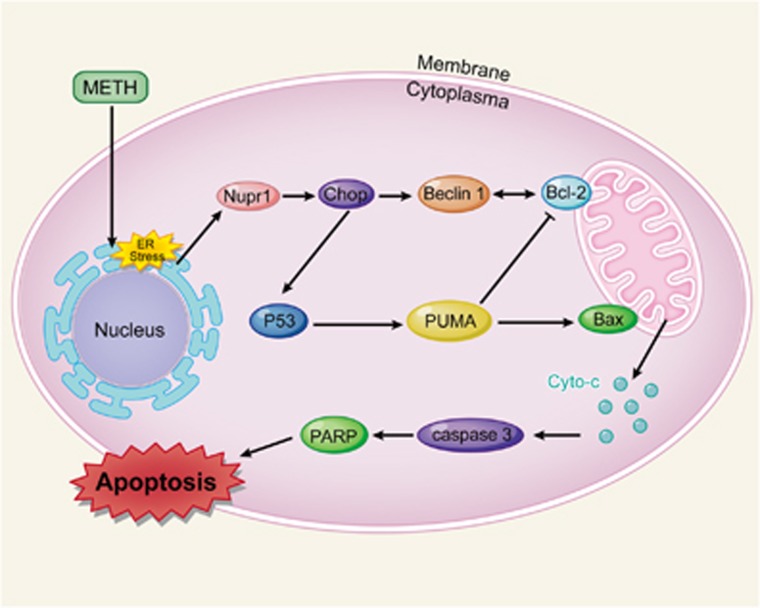
A schematic depicting the role of Nupr1–Chop/P53–PUMA/Beclin1 signaling pathway in METH-induced endothelial cell apoptosis. Briefly, Nupr1 expression is increased following METH treatment. Increased Nupr1 upregulates the expression of Chop. As a transcription factor, Chop upregulates the expression of P53, and P53 raises up PUMA expression, which mediates the mitochondrial apoptosis pathway by changing the ratio of Bax/Bcl-2. Meanwhile, Chop also increases the expression of Beclin1, which forms th eBcl-2/Beclin1 complex, thereby decreasing the dissociative Bcl-2. As a result, the ratio of dissociative Bcl-2 to Bax is also significantly decreased. The increased ratio of Bax/Bcl-2 results in translocation of cyto *c*, an apoptogenic factor, from the mitochondria to cytoplasm and activation of caspase-dependent apoptotic pathways

In this study, we showed that apoptotic effect of METH on vascular endothelial cells (HUVECs and CMECs) is mediated by upregulation of the stress protein Nupr1. Moreover, we provide evidence that Nupr1 upregulation also takes place *in vivo* and this apoptotic effect is associated with increased activation of the Nupr1-regulated apoptotic pathway through Chop. As a transcription factor, Chop upregulates the expression of P53, resulting in upregulation of PUMA, which mediates mitochondrial apoptosis pathway by changing the ratio of Bax/Bcl-2. Chop also increases the expression of Beclin1, which forms Bcl-2/Beclin1 complex and regulates apoptosis. A schematic depicting this novel mechanism of METH-induced endothelial cell apoptosis is provided in Figure 9. The underlying mechanisms about how Beclin1 interacts with Bcl-2 and regulates METH-induced apoptosis need further research. These findings provide insights into the molecular mechanisms of Nupr1-mediated METH-induced apoptosis in endothelial cells. However, our findings are mainly based on *in vitro* experimental results. Future studies using knockout animal models are needed to substantiate the present findings.

## Materials and Methods

### Animal protocol

Healthy adult male Sprague–Dawley (SD) rats (180–220 g, 6–8 weeks old) were purchased from Laboratory Animal Center of Southern Medical University (Guangzhou, China). Animals were housed individually in tub cages with food and water available *ad libitum* on a 12 h light/dark cycle. Animals were acclimated to the animal facility for 1 week before experimentation. Rats were divided randomly into two groups (*n*=5 each group). METH (>99% purity; National Institutes for Food and Drug Control, Guangzhou, China) was dissolved in 0.9% physiological saline. The hyperthermia produced by METH was counteracted using a cooling bath. Rats were injected intraperitoneally (i. p.) with saline or METH (8 injections, 15 mg/kg/injection, at 12 h intervals). This exposure paradigm was chosen based on our and other previous studies to mimic human METH abuse because the measured concentrations of METH in the blood and brain of the METH-treated rats at 1 h after the last injection were in the range of reported blood levels in METH abusers.^5, 45, 46, 47^ All animals survived throughout the study period. Rats were killed at 24 h after the last injection. Hearts were isolated and stored at −86 °C or in 4% paraformaldehyde at 4 °C. All animal procedure was approved by the Institutional Animal Care and Use Committee at the Southern Medical University and in accordance with the latest National Institutes of Health Guide for the Care and Use of Laboratory Animals.

### Cell culture

HUVECs were obtained from the Cell Bank of Shanghai Institute for Biological Science, Chinese Academy of Science and cultured in high glucose DMEM medium containing 10% fetal bovine serum (FBS) (Gibco, Grand Island, NY, USA). Cells were grown in a CO_2_ incubator at 37 °C, with 5% CO_2_ and 95% filtered air. The cells were passaged every 2–3 days. Isolation and identification of rat CMECs were performed as previously described,^48, 49^ with some modifications. Briefly, 2-week-old SD rats were anesthetized by pentobarbital injection. The heart was removed from the rat under sterile condition and then the left ventricular was isolated. After washing with PBS, the epicardium was removed and the remaining myocardium was then minced. The minced tissue was immersed in 0.2% collagenase in 37 °C for 10 min, and then 0.02% trypsin was added for another 10 min to digest the tissue. High glucose DMEM medium with 10% FBS was added to stop digestion, and then the solution was filtered through a 100-μm nylon mesh to remove the undigested tissue. The filtered solution was centrifuged, resuspended in high glucose DMEM medium containing 20% FBS, 0.12% heparin, 1% endothelial cell growth supplement (ECGS) (Sigma, St. Louis, MO, USA), 0.5% streptomycin/penicillin solution (10 000U/ml; Invitrogen, New York, NY, USA) and then cells were seeded on dishes. After 8 h incubation in the cell incubator, the supernatant was discarded, and then the high glucose DMEM medium containing 20% FBS, 0.12% heparin, 1% ECGS and 0.5% streptomycin/penicillin solution was added. Thereafter, the medium was changed every 48 h and the cells were passaged when they reached the exponential phase (70–80% confluence). CMECs were seeded in coverslips and acetylated low-density lipoprotein (15 μg/mg) was added when the cells reached about 80% confluence. After 8 h incubation, the coverslip was washed with PBS and fixed with 4% paraformaldehyde solution. The antibodies for CD31 (mouse, 1:100; Santa Cruz, Dallas, TX, USA) and fluorescein (Cy3)-conjugated rabbit anti-mouse IgG (1:50; DingGuo, Dalian, China) were used together with 4′,6′-diamidino-2-phenylindole (DAPI) nuclear labeling. Incubation periods with blocking buffer, primary antibody, and secondary antibody were 30 min at room temperature, overnight at 4 °C and 1 h at room temperature, respectively. Microphotographs were taken using a fluorescence microscopy (A1+/A1R+ Nikon, Tokyo, Japan). Confluent CMECs displayed typical ‘flagstone' morphology. The representative immunofluorescence image demonstrated that the purity of rat primary dissociated CMECs is more than 95% (Supplementary Figure S8).

### Western blot analysis

HUVECs and CMECs were seeded on six-well plates at a density of 3 × 10^5^/well. Cells were exposed to saline vehicle or METH (1.25 mM for HUVECs and 0.5 mM for CMECs) for 24 h. These concentrations were selected based on other METH *in vitro* toxicity studies^11, 50^ and the LC_25_ of METH in HUVECs and CMECs (Supplementary Table S1). After exposure to different time points, HUVECs and CMECs were lysed in RIPA buffer (Sigma) at 4 °C for 30 min. Cell lysates were stored at −86 °C. The total protein concentration was determined using the Bradford method. The samples were then separated with SDS-polyacrylamide gel electrophoresis and electroblotted onto polyvinylidenedifluoride membranes. After blocking with TBST-buffered saline solution containing 5% dry milk for 1.5 h, the membranes were incubated overnight at 4 °C or at 25 °C for 2 h with primary antibodies, including rabbit polyclonal anti-Nupr1 (1:1000; BioVision, Milpitas, CA, USA), rabbit polyclonal anti-Chop, rabbit polyclonal anti-Beclin1, rabbit polyclonal anti-Puma, rabbit polyclonal anti-Bax, rabbit polyclonal anti-Bcl-2, rabbit polyclonal anti-cyto *c*, rabbit polyclonal anti-caspase-3, rabbit polyclonal anti-PARP (all in 1:1000 and from the Cell Signaling Technology, Danvers, MA, USA), rabbit P53 (1:500; ABclonal Biotech Co., Ltd, College Park, MD, USA) and rabbit polyclonal anti-*β*-actin (1:2000; Cell Signaling Technology). Thereafter, membranes were incubated with corresponding horseradish peroxidase-conjugated secondary antibodies at 25 °C for 1 h. Membranes were developed with Chemiluminescence ECL^Plus^ western blotting detection reagents. Proteins of interest were quantified based on pixel density with the Gel-Pro analyzer software (Media Cybernetics, Inc., Rockville, MD, USA) and then normalized to a correspondent *β-*actin loading control prior to statistical analyses.

### Annexin V apoptosis staining

HUVECs and CMECs were seeded on six-well plates at a density of 2 × 10^6^/well. Cells were exposed to saline vehicle or METH (1.25 mM for HUVECs and 0.5 mM for CMECs) for 24 h. Cells were centrifuged to remove the medium, washed twice with 4 °C PBS, and stained with Annexin V-FITC and propidiumiodide (Keygen, Nanjing, China) according to the Annexin V apoptosis detection kit instructions. The percentage of apoptotic cells was quantified using a flow cytometry system (FACSCalibur; BD Biosciences, San Jose, CA, USA).

### TUNEL staining

HUVECs and CMECs (5 × 10^5^/well) were exposed to METH (1.25 mM for HUVECs and 0.5 mM for CMECs) for 24 h. DNA fragmentation was visualized using a fluorometric TUNEL system for apoptotic cells (Roche Applied Science, Indianapolis, IN, USA) according to the manufacturer's instructions. Briefly, HUVECs and CMECs were fixed in 4% paraformaldehyde in ice-cold PBS (pH7.4) at room temperature for 15 min, incubated with fluorescein-conjugated TdT enzyme at 37 °C for 1 h in the dark and then mounted with DAPI for nuclear counter staining. For frozen tissues, 5 *μ*m tissue sections were sliced using a freezing microtome (CM1900; Leica, Wetzlar, Germany). The tissue sections were placed on a Fisher Superfrost slide and dried overnight at room temperature (RT). The slides were fixed by immersion in cold acetone (−20 °C) for 2 min and then air-dried at RT. The pretreatment of tissue sections (including deparaffinizing, rehydration, retrieval with reconstituted proteinase K, equilibration and slide washing) were performed according to the manufacturer's instructions. Tissue slides were incubated with Nucleotide Mix and rTdT buffer solution at 37 °C for 60 min to allow sufficient reaction. The reaction was terminated by adding 2 × SSC solution (a concentrated solution of sodium chloride–sodium citrate in distilled/deionized water), followed by 15 min incubation. The control incubation buffer was prepared without the rTdT enzyme; all other steps were similar. Samples were stained with DAPI to determine the total number of nuclei. Cross-sections were photographed (× 20 and × 40 objectives) using a fluorescence microscope (ECLIPSE 80i; Nikon). Both TUNEL- and DAPI-positive cells were counted. Data are presented as the TUNEL index, which was calculated based on the total number of TUNEL-positive cells.

### Measurement of mitochondrial membrane potential

The mitochondrial membrane potential was determined using JC-1 kit (C2006; Beyotime, Haimen, China). HUVECs were seeded (2 × 10^5^ cells/well) in six-well culture plates. After 24 h exposure with vehicle or METH (1.25 mM), the supernatant was removed and cells were washed with PBS and then maintained in JC-1-containing DMEM medium for 20 min at 37 °C in the dark. Thereafter, cells were washed twice with buffer solution (4 °C) and analyzed with flow cytofluorometry.

### Double immunofluorescence labeling

To determine Nupr1 expression level in cardiac endothelial cells, we performed double immunofluorescence labeling on frozen sections of adult rat hearts as described before.^51^ For immunolabeling, all incubation solutions were prepared using PBS supplemented with 10% normal goat serum and 0.05% Triton X-100. The antibodies used were CD31 (mouse, 1:100; Santa Cruz), Nupr1 (rabbit, 1:100; Santa Cruz), fluorescein (FITC)-conjugated rabbit anti-mouse IgG (1:50; DingGuo), fluorescein (Cy3)-conjugated sheep anti-rabbit IgG (1:50; DingGuo). These antibodies were used together with DAPI nuclear labeling. The frozen tissue sections were incubated with blocking buffer (5% skim milk) for 30 min at RT, with the primary antibody overnight at 4 °C, and then with the secondary antibody for 1 h at RT. Microphotographs were taken using fluorescence microscopy (A1+/A1R+ Nikon). All digital images were processed using the same settings to improve the contrast.

### siRNA and transfection

siRNA sequences that target Nupr1 (2 pieces), Chop (4 pieces), P53 (4 pieces), Puma (4 pieces) or Beclin1 (4 pieces) were designed by Shanghai GenePharma Co. Ltd (Shanghai, China), as shown below: siNupr1#1 (Human, 5′-CAGACAAAGCGUUAGGAGA-3′), siNupr1#2 (Human, 5′-CAGAGACAGACAAAGCGUU-3′), siChop#1 (Human, 5′-GGGAUACCAUGCAACAUAA-3′), siChop#2 (Human, 5′-CUAGAAAUCUGUUGCUAUG-3′), siChop #3 (Rats, 5′-CCTGTCCTCAGATGAAATT-3′), siChop #4 (Rats, 5′-CCAGATTCCAGTCAGAGTT-3′), siP53 #1 (Human, 5′-UCAAAUCAUCCAUUGCUTT-3′), siP53 #2 (Human, 5′-UUACAUCUCCCAAACAUTT-3′), siP53 #3 (Rat, 5′-UACAUUAUUUCAUUAAATT -3′), siP53 #4 (Rat, 5′-AAAACCUUAAAAUCUAATT-3′), siPuma #1 (Human, 5′-GGGUCCUGUACAAUCUCAUTT-3′), siPuma #2 (Human, 5′-CGAGAUGGAGCCCAAUUAGTT-3′), siPuma #3 (Rat, 5′-GCGGAGACAAGAAGAGCAATT-3′), siPuma #4 (Rat, 5′-GUCAUGUAUAAUCUCUUCATT-3′), siBeclin1 #1 (Human, 5′-CUGGACACGAGUUUCAAGATT-3′), siBeclin1 #2 (Human, 5′-GUGGAAUGGAAUGAGAUUATT-3′), siBeclin1 #3 (Rat, 5′-CAGGAGAGGAGCCAUUUAUTT-3′), siBeclin1 #4 (Rat, 5′-GUCCCUGACAGACAAAUCUTT-3′), siRNAs were dissolved in DEPC water at a concentration of 20 *μ*M. siRNA transfection was performed according to the instructions of Lipofectamine 2000 Transfection Reagent kit from Invitrogen. Briefly, the siRNA for each gene of interest and the corresponding control siRNA were separately mixed with Lipofectamine 2000, vortexed for 20 s, and then incubated at RT for 30 min prior to use. HUVECs and CMECs were seeded on six-well plates at a density of 2 × 10^6^/well. siRNA mixture was added gently and slowly, and then 1 ml complete medium was added in each well. After 6 h incubation, all supernatant was discarded and then 2 ml complete medium was added in each well.

### Lentivirus production and infection of CMECs cell

The shRNA synthesis was based on a previous study.^5^ Briefly, the shRNA sequence targeting Nupr1 (5′-GCAACCTGTAAACATAGAG-3′ and 5′-GCCTGGCCCAATCTTATGT-3′) was cloned into pGC-LV vector. pGC-LV-shNupr1, pHelper 1.0, and pHelper 2.0 were cotransfected into HEK293FT cells. LV-shNupr1 was harvested with 1 × 10^9^ transducing units per milliliter and LV-GFP was used as the reference control virus. Viral supernatants were collected at 72 h, centrifuged at 4500 × *g* for 5 min, filtered through a 0.22 *μ*m filter, and incubated with CMECs and polybrene (5 *μ*g/ml). After 24 h of viral transduction, cells were given fresh growth media and the cells were passaged when they reached the exponential phase.

### Co-immunoprecipitation assay and immunoblotting analysis

After exposure to METH (1.25 mM for HUVECs and 0.5 mM for CMECs) or vehicle for 24 h, cells were washed with ice-cold lysis buffer containing protease inhibitor mix (Sigma). The lysates were incubated with Beclin1 antibody for 1 h and then with protein A/G-agarose beads at 4 °C for 12 h. The immunoprecipitates were pelleted, washed and subjected to immunoblotting using Bcl-2 or Beclin1 antibody as described above.

### Statistical analysis

Data given in the text are expressed as mean± standard deviation (S.D.) of at least three independent replicates. Statistical analysis was performed using one-way ANOVA followed by LSD *post hoc* analysis or independent-samples *t*-test (as appropriate) using the scientific statistic software SPSS version 19.0 (SPSS Inc., Chicago, IL, USA). The value of *P*<0.05 was considered statistically significant.

## Abbreviations

BBB: blood–brain barrier
CMECs: cardiac microvascular endothelial cells
cyto *c*: cytochrome *c*
CHOP: C/EBP homologous protein
ERS: endoplasmic reticulum stress
FBS: fetal bovine serum
HUVECs: human umbilical vein endothelial cells
METH: methamphetamine
MMP: mitochondrial transmembrane potential
PERK: PKR-like endoplasmic reticulum kinase
PUMA: p53 upregulated modulator of apoptosis
Nupr1: nuclear protein 1
TUNEL: terminal deoxynucleotidyl transferase (TdT)-mediated UTP nick end labeling

## Supplementary Material

Supplementary Informations
