# Supplementary material for: Nupr1/Chop signal axis is involved in mitochondrion-related endothelial cell apoptosis induced by methamphetamine
Source: Cell Death Dis. 2016 Mar 31;7(3):e2161–. doi: 10.1038/cddis.2016.67 (PMC4823965; doi:10.1038/cddis.2016.67)
Supplement: Supplementary Informations [file cddis201667x1.doc]

**Nupr1/Chop signal axis is involved in mitochondrion-related endothelial cell apoptosis induced by methamphetamine**

Dunpeng Cai^1^, Enping Huang^1^, Baoyin Luo^1^, Yaguang Yang^1^, Chao Liu^2^, Zhoumeng Lin^3^, Wei-Bing Xie^1§^, Huijun Wang^1§^

1 Department of Forensic Medicine, School of Basic Medical Science, Southern Medical University, Guangzhou 510515, People’s Republic of China; 2 Guangzhou Forensic Science Institute, Guangzhou 510030, People’s Republic of China; 3 Institute of Computational Comparative Medicine, Department of Anatomy and Physiology, College of Veterinary Medicine, Kansas State University, Manhattan, KS 66506, USA

**Running title**: Nupr1/Chop and METH-induced EC apoptosis

§ Corresponding author at: Department of Forensic Medicine, School of Basic Medical Science, Southern Medical University, Guangzhou 510515, People’s Republic of China. Telephone: +86-2062789044. E-mail addresses: [hjwang711@yahoo.cn](mailto:hjwang711@yahoo.cn) (Huijun Wang), [xieweib@126.com](mailto:xieweib@126.com) (Wei-Bing Xie).

**Materials and methods**

**1. LC25 and LC50 of METH in HUVECs cells and CMECs cells**

Cell counting kit-8 (Dojindo, Japan) was used to determine the LC25 and LC50 values of the cells according to the manufacturer's instructions. Briefly, 10uL Cell Counting Kit (CCK8) reagent was added to each well (5×10^3^ cells/well) after 24h exposure to different concentrations of METH (0, 0.2, 0.4, 0.6, 0.8, 1.0, 1.2, 1.4, 1.6, 1.8, 2.0, 2,2, 2.4, 2.6mM) (n=6). Then the 96-well plate was incubated in the cell incubator for 2h. Enzyme standard instrument was used to measure the absorbance values at 450 nm.

**Table 1** The values of LC25 and LC50 of METH in HUVECs

cell line and CMECs cell line.

|  | LC25 | LC50 |
| --- | --- | --- |
| HUVECs | 1.54mM | 2.37mM |
| CMECs | 0.67mM | 1.13mM |

**2. Purity determination of primary cultured CMECs.**

CMECs were seeded in coverslips. Acetylated low density lipoprotein (15μg/mg) was added when the cells reached about 80% confluence. After 8h incubation with acetylated low density lipoprotein, the coverslip was washed with PBS, fixed with 4% paraformaldehyde solution. The antibody against CD31 (mouse, 1:100, Santa Cruz) and fluorescein (Cy3)-conjugated rabbit anti-mouse IgG (1:50, DingGuo, China) were used together with DAPI nuclear labeling. The coverslip was blocked with blocking buffer, incubated with the primary antibody, and then submerged in the secondary antibody for 30min at room temperature, overnight at 4°C, and 1h at room temperature, respectively. Microphotographs were taken using a fluorescence microscopy (A1+/A1R+, Nikon, Tokyo, Japan).

**Supplementary Figures**

**
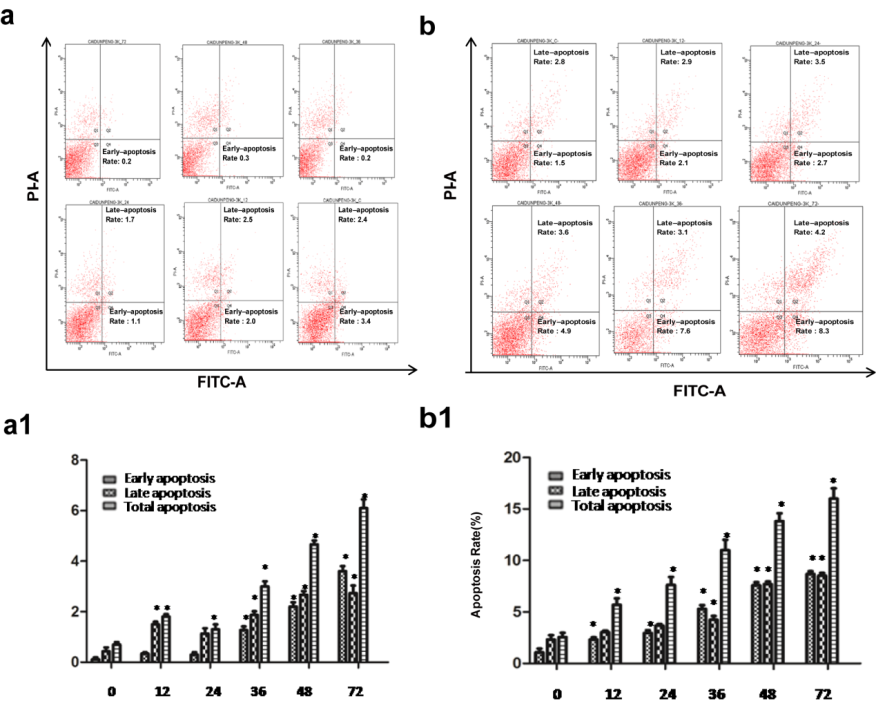
**

**Suppl. Fig. 1 Detection of Apoptosis induced by METH in HUVECs and CMECs cells.** a, HUVECs cells were treated with 1.25mM METH for indicated time (0, 12, 24, 36, 48,72 h). b, CMECs cells were exposed to 0.5mM METH for indicated time (0, 12, 24, 36, 48, 72h). Cell apoptosis was determined using a flow cytometry system.

**
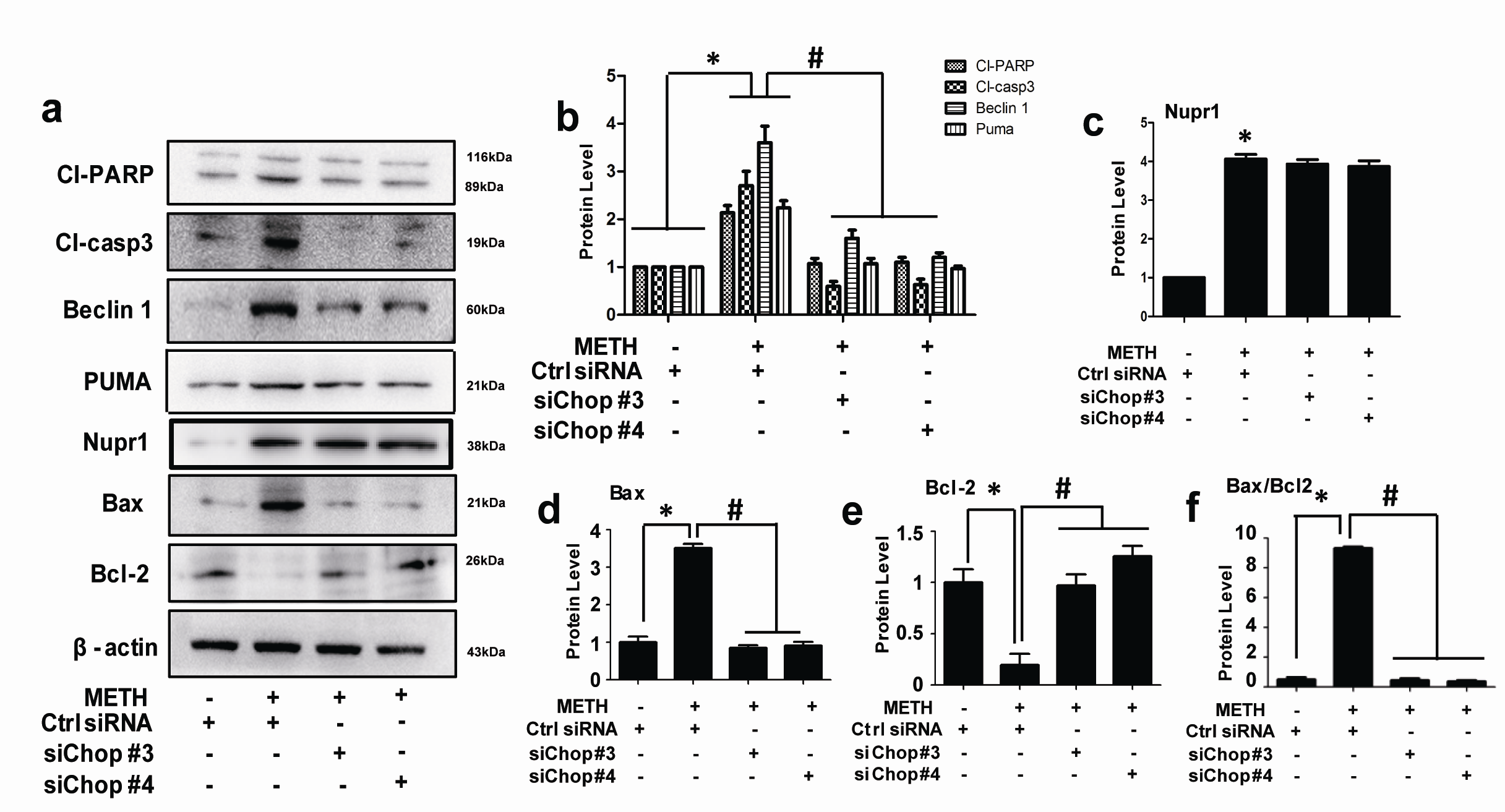
**

**Suppl. Fig. 2 Chop is involved in METH-induced apoptosis in CMEC cells**. a, CMECs cells were transfected with siRNAs targeting Chop or ctrl siRNA for 48h followed by METH (0.5mM) treatment for 24h. Protein samples were collected. Western blot and quantitative analyses were performed to evaluate the expression of cleaved-PARP (Cl-PARP), cleaved-caspase3 (Cl-casp3), Beclin1, PUMA, Nupr1, Bax and Bcl-2.

**
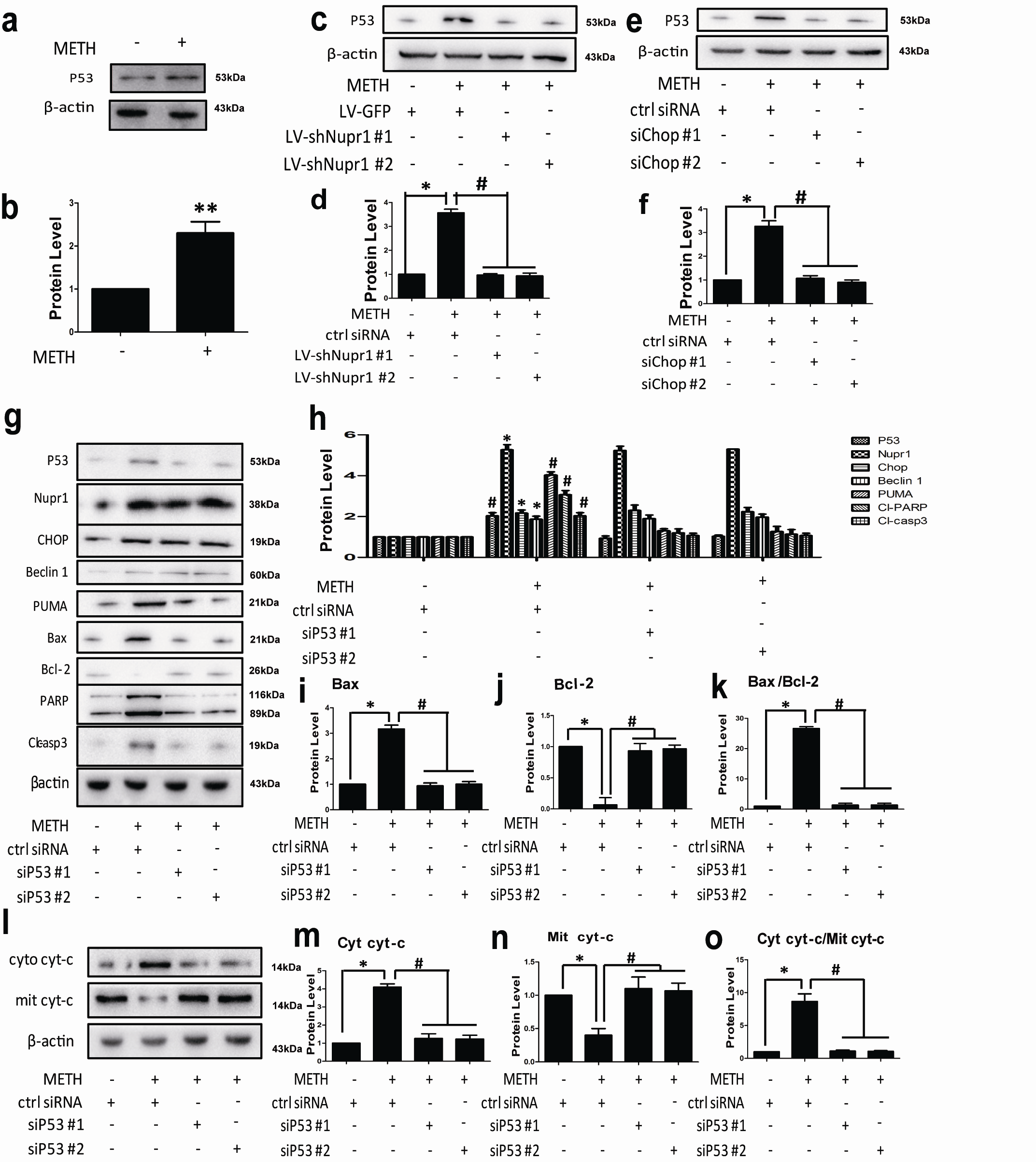
**

**Suppl. Fig. 3 P53 is involved in Nupr1-Chop axis-mediated mitochondrial apoptotic signaling pathways caused by METH in endothelial cells.** a, CMECs cells were exposed to METH (0.5mM) for 24h. c, CMECs cells were transfected with LV-shNupr1 targeting Nupr1 or LV-GFP for 48h followed by METH (0.5mM) treatment for 24h. e, CMECs cells were transfected with siRNAs targeting Chop or ctrl siRNA for 48h followed by METH (0.5mM) treatment for 24h. g, CMECs cells were transfected with siRNAs targeting P53 or ctrl siRNA for 48h followed by METH (0.5mM) treatment for 24h.Western blot (a, c, e, g) and quantitative analyses (b, d, f, h, i, j) were performed to evaluate the expression of P53, Beclin1, Bax, Bcl-2, Chop, Nupr1, cleaved-PARP (Cl-PARP), and cleaved-caspase-3 (Cl-casp3). The protein levels of cytosolic and mitochondrial cyto c (l, m, n) were measured using Western blot analyses. The Bax/Bcl-2 (k) and cyto cyt-c/mit cyt-c (o) ratios were calculated. Data are presented as mean±SD (n = 3). *p < 0.01 vs. the saline vehicle-treated ctrl group, **p < 0.01 vs. scrambled + METH group (one-way ANOVA).

**
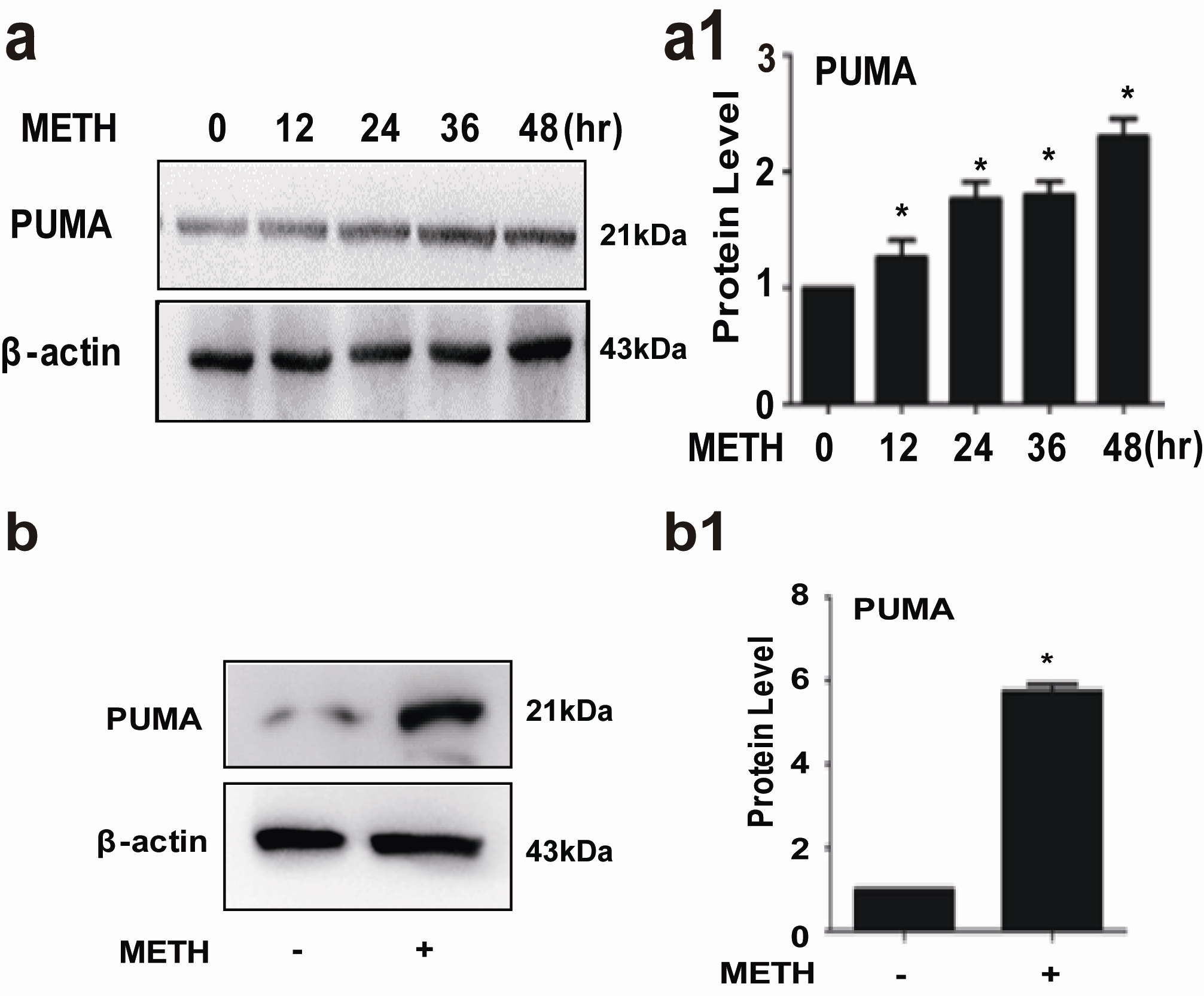
**

**Suppl. Fig. 4 PUMA expression level is increased by METH exposure in both HUVECs and CMECs.** HUVECs cells were treated with 1.25mM METH for indicated time (0, 12, 24, 36, 48h) and CMECs cells were exposed to 0.5mM METH for 24h. Protein samples were collected. Western blot (a and b) and quantitative analyses (a1 and b1) were performed to evaluate the expression of PUMA.

**
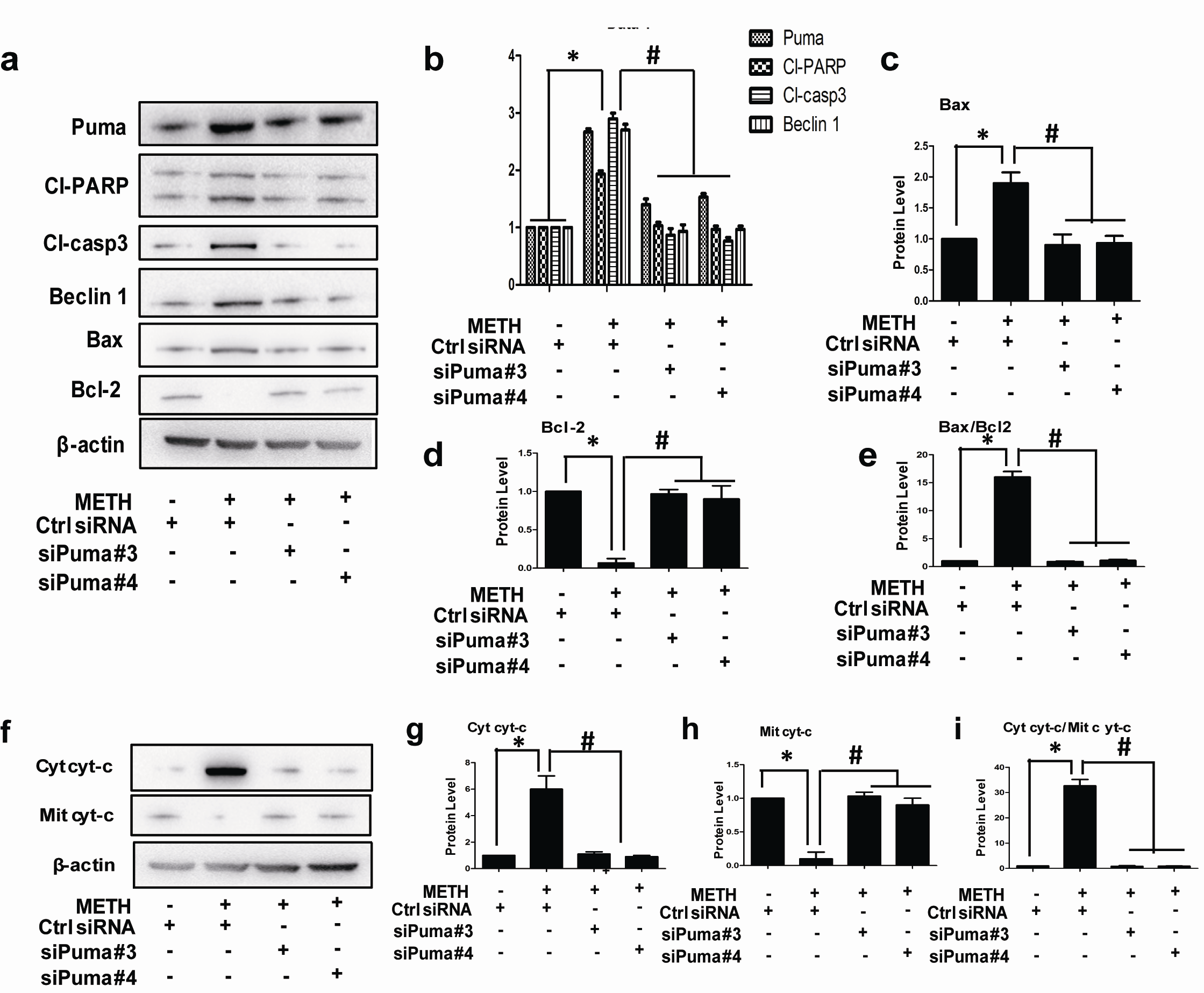
**

**Suppl. Fig. 5 Puma participates in the apoptosis induced by METH in CMECs cells.** CMECs cells were transfected with siRNAs targeting Puma or control siRNA for 48h followed by METH (0.5mM) treatment for 24h. Western blot (a, f) and quantitative analyses (b-e, g-i) were performed to evaluate the expression of Puma, cleaved-PARP (Cl-PARP), cleaved-caspase3 (Cl-casp3), Beclin1, Bax, Bcl-2, Bax/Bcl-2 ratio, cytosolic cyto c (Cyt cyt-c), and mitochondrial cyto c (Mit cyt-c), and Cyt cyt-c/Mit cyt-c ratio.

**
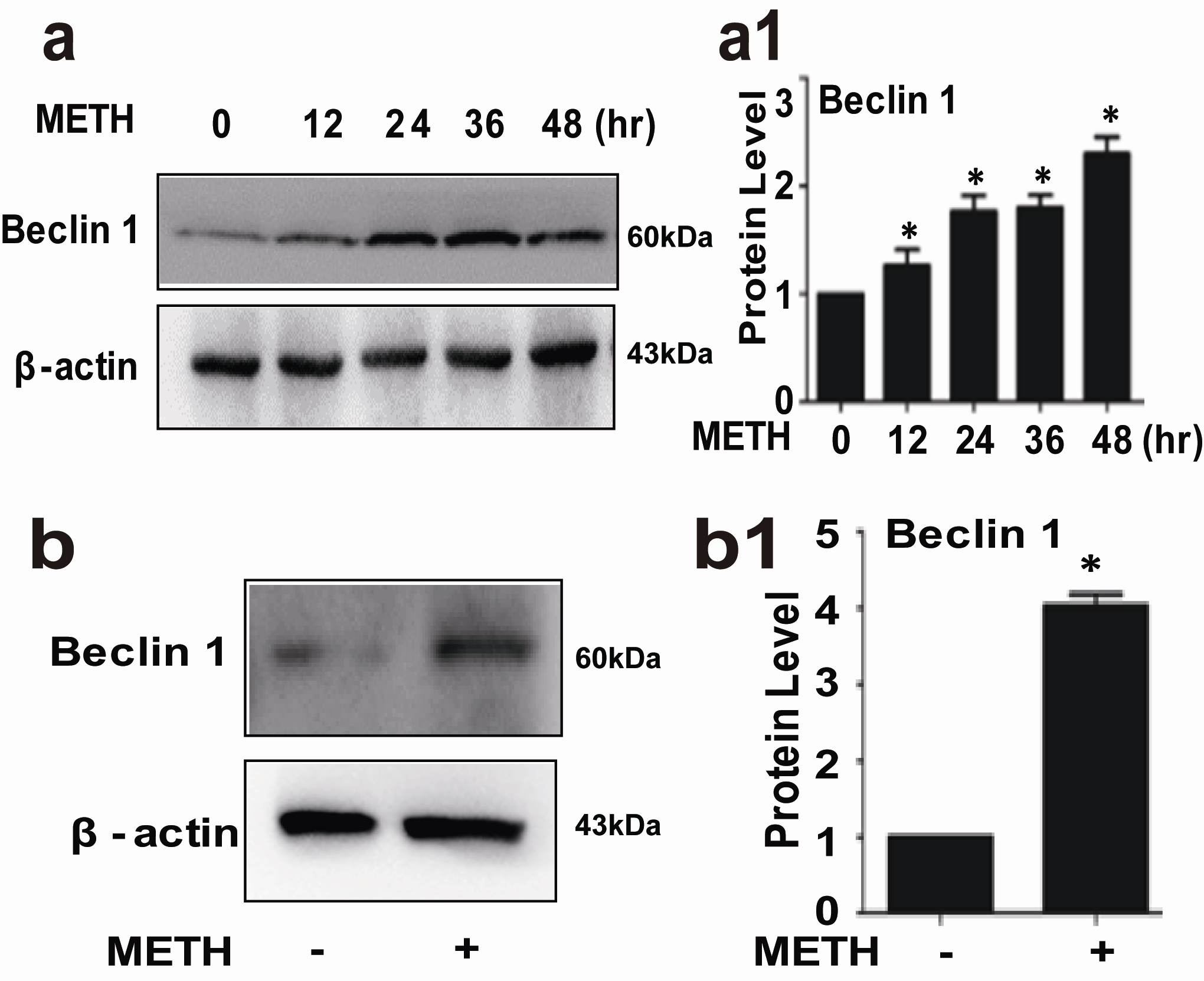
**

**Suppl. Fig. 6 METH exposure increases Beclin1 expression in both HUVECs and CMECs.** HUVECs cells were treated with 1.25mM METH for indicated time (0, 12, 24, 36, 48h) and CMECs cells were exposed to 0.5mM METH for 24h. Western blot (a and b) and quantitative analyses (a1 and b1) were performed to evaluate the expression of Beclin1.

**
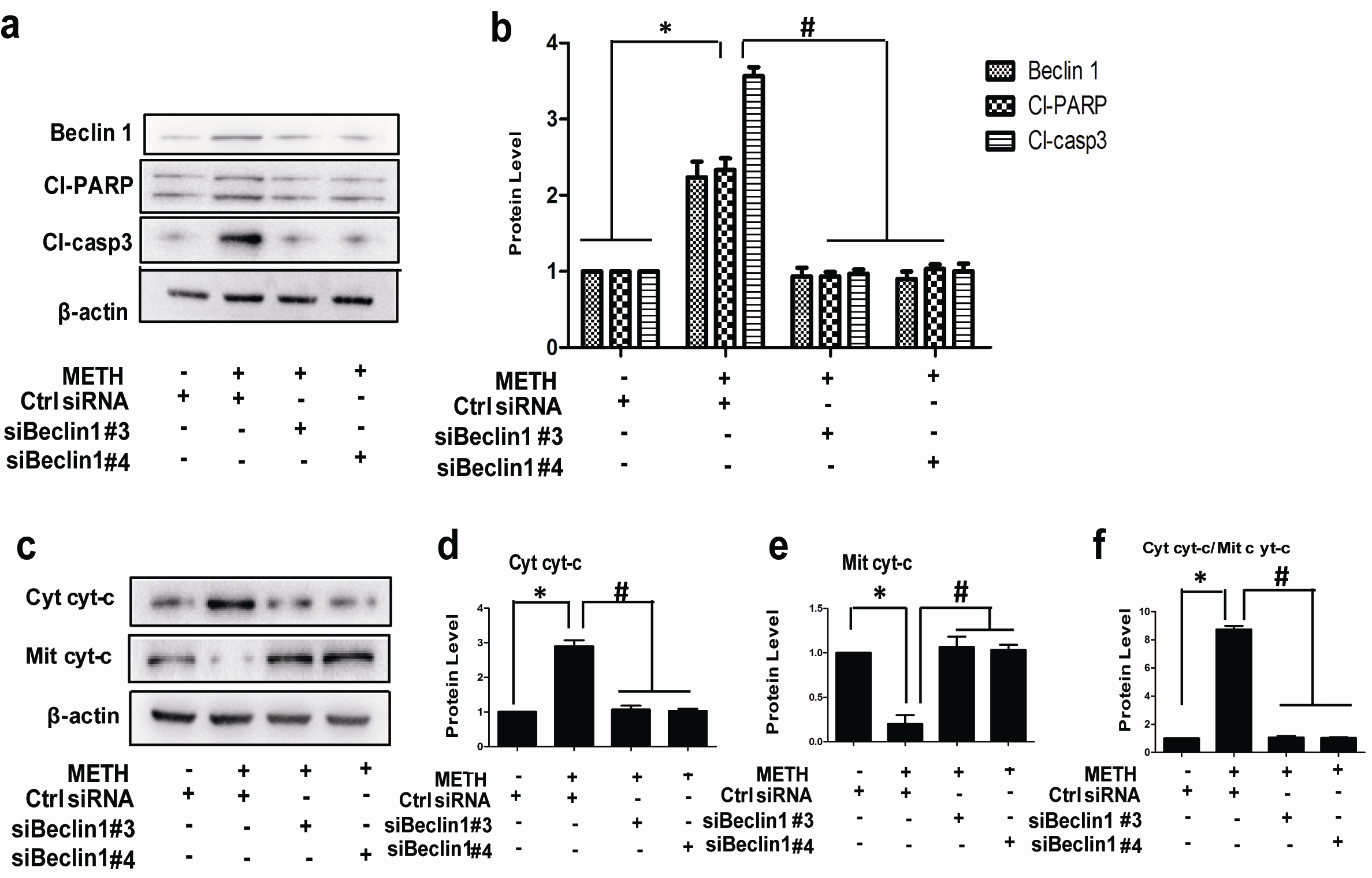
**

**Suppl. Fig. 7 Beclin 1 participates in the apoptosis induced by METH in CMECs cells.** CMECs cells were transfected with siRNAs targeting Beclin1 or ctrl siRNA for 48h followed by METH (0.5mM) treatment for 24h. Western blot (a, c) and quantitative analyses (b, d-f) were performed to evaluate the expression of Beclin 1, cleaved-PARP (Cl-PARP), cleaved-caspase3 (Cl-casp3), cytosolic cyto c (Cyt cyt-c), mitochondrial cyto c (Mit cyt-c), and Cyt cyt-c/Mit cyt-c ratio.

**
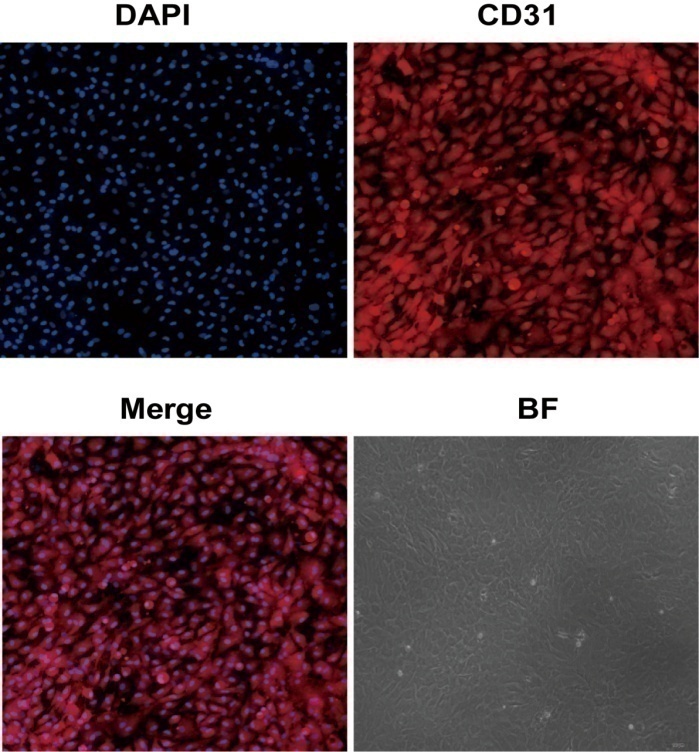
**

**Suppl. Fig. 8 Purity determination of primary cultured CMECs.** DAPI labeling represents the nuclear. CD31 labeled with CY-3 represents the specific marker of vascular endothelial cells, which shows that the purity of primary cultured CMECs is more than 95%. BF represents bright field.
